# Hidden value

**DOI:** 10.7554/eLife.50543

**Published:** 2019-08-20

**Authors:** Brooke Morriswood, Oliver Hoeller

**Affiliations:** 1 Department of Cell and Developmental BiologyUniversity of WuerzburgWuerzburgGermany; 2 Freelance science illustratorSan FranciscoUnited States

**Keywords:** point of view, metrics, science policy, science funding, research evaluation

## Abstract

The fact that it is difficult to evaluate and compare the outputs of individual researchers might actually be good for science.

Metrics are now ubiquitous in science – for journals, for the papers within them, and for the authors of those papers. One reason why metrics have become attached to individual researchers is that various bodies need to assign value to a person's research – for example, when allocating research grants, or making decisions about promotion and tenure. This yearning for a simple measure of comparison is understandable. Like any voyage into the unknown, research is an inherently risky endeavor, and we crave a means of placing a value on the bounty we obtain from the beyond. Pirates and traders could compare their piles of treasure, but what should scientists count?

In this sense, the tension around metrics for individuals is just another iteration of an older debate – whether evaluations are best made based on either expert intuition or cold multi-parametric calculation. The current appeal of metrics reflects a concerning but unavoidable reality: nowadays intuition is of almost no use at all. This is because it has become almost impossible to judge the value of work conducted outside our own research niche.

The debate about metrics is basically a response to the fact that everybody has become specialized to a degree that makes a kettle look like a Swiss Army knife. Consequently, it is extremely hard to judge the quality and value of work done outside our own little scientific habitat. Technical specialization, improved resolution, and thematic speciation have turned scientific research from a mushrooming field into a field of mushrooms, with each sub-community focused squarely on the area under its cap. As such, all the motivation behind challenging old metrics or championing new ones is driven by our recognition that (a) judging value is difficult, and (b) that we’d be better off with either a better metric or a better way of defining the value of what we do.

But are we missing something here? What if it is actually an advantage that we are unable to assign value?

The unique and special thing about science – which we should cherish – is not just the control of our own freedom (which is important) but also the way it is funded. In most businesses the top priority is to turn a profit, and the stress experienced by workers comes from ensuring that their company does so. No profit, no value. As soon as value can be sharply defined, it allows everything to be refocused around market forces. And the application of market forces means that something that is unlikely to be profitable will not be pursued, regardless of how interesting or fun it might be. If this were truly the case in science, we would probably have even greater hegemony than we do now – and we're already at a point where journals debate if *C. elegans* work receives enough citations, and where drug development for neglected tropical diseases is disincentivized.

In science, any profit as a result of taxpayer investment is generally far downstream, and the overriding motivation is curiosity rather than potential future revenue. Investing public money in research generates a return in the form of knowledge, but actual profits lie off in the distance (and often require further investments, which can be substantial in some cases, such as clinical trials).

It means that in academia, our stress comes from seeking funding, but everyone knows not to expect a guaranteed payoff. Of course, if you get no results then you will still struggle for funding, but the punch line is that our inability to precisely assign value actually enables the persistence of less mainstream research topics, and sustains the diversity of the enterprise as a whole.

**Figure 1:**
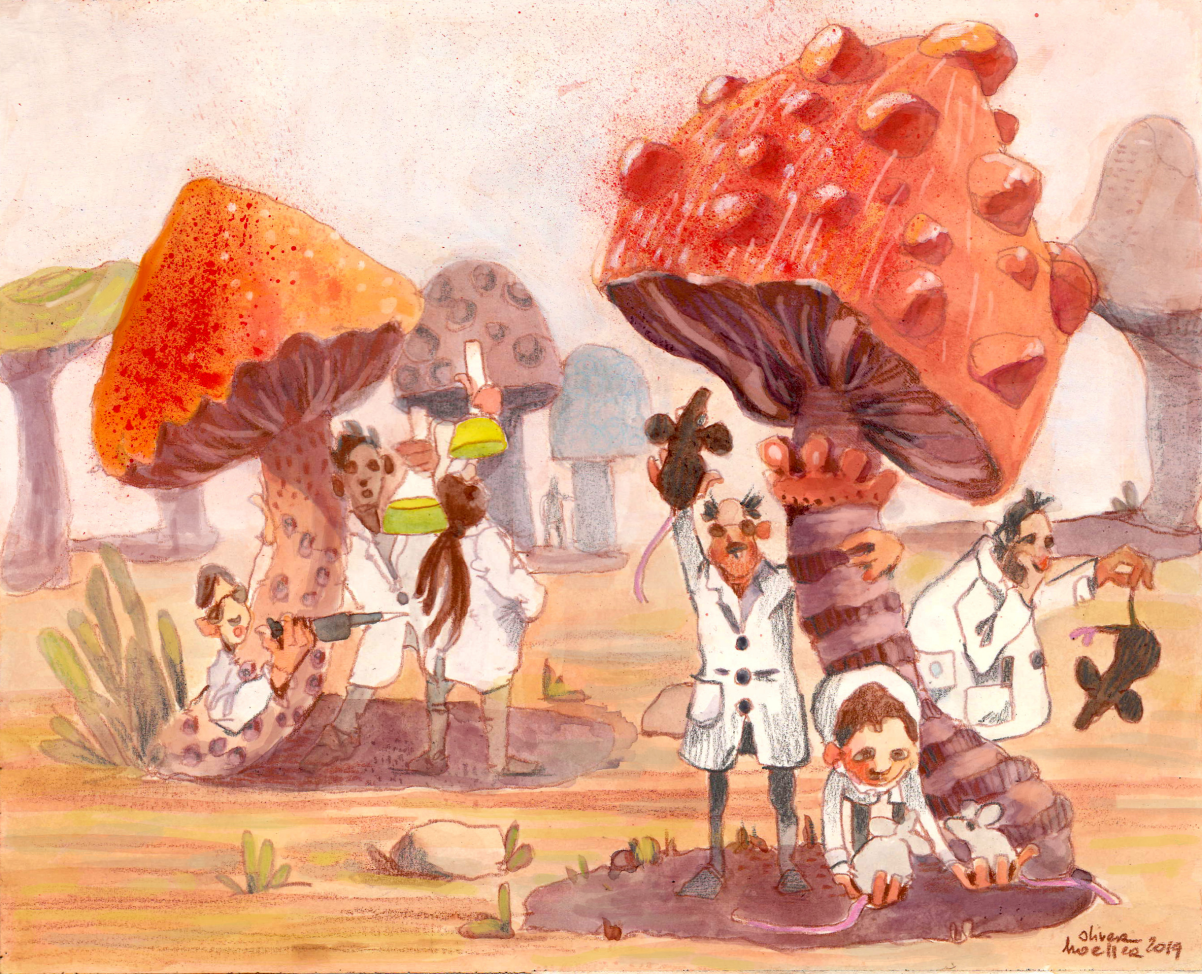
It has become almost impossible for researchers to judge the value of work conducted outside their own research niche.

Illustration: Oliver Hoeller

Coalesce around a single metric and you may end up with the same situation as occurred in the UK pop charts in 2017 – Ed Sheeran occupying 16 places in the top 20. Sheeran’s bland, beatific dominance likely mirrors the kind of consolidation that would follow if science ever actually agreed on a single definition of value. Nothing wrong in terms of quality, and enormously popular, but having all chart positions occupied by one organism cannot be good for everyone.

It is still tough working outside the mainstream, and it is harder to get the work into prestige journals (if you care about that kind of thing), but a career-long existence is still possible. Nobody would ever have suspected that something as esoteric as research into bacterial RNAs would ultimately lead to the CRISPR revolution, but this is merely the latest of a long list of examples – a list that shows the treasures that can be unearthed by looking outside the glare of fashion’s lamp.

The ultimate value of science remains hidden and it’s the search – not the sale – that is important. Let’s keep it that way.

